# Co-delivery of doxorubicin and hydroxychloroquine via chitosan/alginate nanoparticles for blocking autophagy and enhancing chemotherapy in breast cancer therapy

**DOI:** 10.3389/fphar.2023.1176232

**Published:** 2023-05-09

**Authors:** Hui Zhang, Qingwen Xue, Zihan Zhou, Ningning He, Shangyong Li, Cheng Zhao

**Affiliations:** ^1^ Department of Abdominal Ultrasound, The Affiliated Hospital of Qingdao University, Qingdao, Shandong, China; ^2^ School of Basic Medicine, Qingdao Medical College, Qingdao University, Qingdao, China; ^3^ Sino Genomics Technology Co., Ltd, Qingdao, China

**Keywords:** co-delivery, combination chemotherapy, nanoparticles, breast cancer, autophagy

## Abstract

Breast cancer (BC) is the most common malignancy in women worldwide, and the standard treatment is chemotherapy or radiotherapy after surgery. In order to reduce the side effects of chemotherapy, various nanoparticles (NPs) have been discovered and synthesized, which has become a promising treatment for BC. In this study, a co-delivery nanodelivery drug system (Co-NDDS) was designed and synthesized with 2,3-dimercaptosuccinic acid (DMSA) coated Fe_3_O_4_ NPs as core encapsulated into chitosan/alginate nanoparticles (CANPs) shell, doxorubicin (DOX) and hydroxychloroquine (HCQ) as loading drugs. Smaller NPs carrying DOX (FeAC-DOX NPs) were loaded into larger NPs containing HCQ (FeAC-DOX@PC-HCQ NPs) by ionic gelation and emulsifying solvent volatilization methods. The physicochemical properties of this Co-NDDS were characterised, followed by *in vitro* studies of the anticancer effects and mechanisms using two different BC cell lines, MCF-7 cells and MDA-MB-231 cells. The results indicated that the Co-NDDS showcases exemplary physicochemical qualities and encapsulation capacity, facilitating accurate intracellular release through pH-sensitive attributes. Importantly, NPs can significantly increase the *in vitro* cytotoxicity of co-administered drugs and effectively inhibit the autophagy level of tumour cells. The Co-NDDS constructed in this study provides a promising strategy for the treatment of BC.

## 1 Introduction

Breast cancer (BC) is one of the most common malignancies in the world, and its increasing morbidity and mortality rates threaten the health and even the lives of millions of patients every year (Global Burden of Disease Cancer et al., 2018; Sun et al., 2018). At present, the treatment of BC is a multimodal strategy combining surgery, chemotherapy, radiotherapy and hormone therapy. The main clinical treatment is post-operative chemotherapy, where patients are given effective chemical anti-tumour cytotoxic drugs to combat the tumour. Chemotherapy prevents the unrestricted growth of tumour cells and inhibits the metastatic spread of tumours to a certain extent (Yang et al., 2021; O'Grady and Morgan, 2021). Despite its efficiency, single chemotherapy is associated with a range of serious side effects, such as a compromised immune system, liver and kidney issues, a reduction in white blood cells, and even the possibility of tumour cells becoming resistant to chemotherapy drugs, making it a challenging treatment choice. In order to reduce the toxic side effects of the drugs on the whole body, enhance the sensitivity of tumour cells to the drugs and thus effectively avoid drug resistance, some combination chemotherapy regimens have emerged (Li et al., 2017a; Hu et al., 2018).

Recent studies (Aiello et al., 2021) have demonstrated a growing interest in the loading of anti-cancer drugs into nanodelivery drug systems (NDDSs) as a means of treatment. NDDS have made notable advances when used in combination with anti-cancer drugs, resulting in enhanced therapeutic efficacy and fewer toxic side effects (Farooq et al., 2019; Huang et al., 2019). As early as 1995, researchers announced the first liposome-based nanomedicine, Doxil^®^, for the treatment of tumours (Irvine and Dane, 2020). Based on the above background, co-delivery NDDS (Co-NDDS) were gradually developed, where at least two anticancer drugs with different physicochemical and pharmacological properties are loaded into a co-delivery system for clinical combination chemotherapy (Qi et al., 2017). Wang *et al.* loaded the anticancer drugs doxorubicin (DOX) and paclitaxel (PTX) onto co-delivered polyethylene glycol poly (lactic-co-glycolic acid) (PEG-PLGA) nanoparticles (NPs) which were co-delivered to increase the effectiveness of free DOX and PTX combination chemotherapy for non-small cell lung cancer (Wang et al., 2011). A similar synergistic effect was obtained by Xiao *et al.* in the evaluation of biodegradable polymers co-releasing DOX and oxaliplatin to enhance anticancer therapy (Xiao et al., 2012).

As a common chemotherapeutic agent for BC, DOX is an antibiotic with broad-spectrum anti-tumour activity (Molinaro et al., 2020). Its main mechanism of action is to block the synthesis of nucleic acids embedded in DNA, thereby inducing DNA damage and apoptosis in cancer cells, and has a strong cytotoxic effect (Bao et al., 2012; Tun et al., 2019). In recent years, due to the fact that DOX alone can lead to an increased autophagy level of tumour cells and their resistance to chemotherapeutic drugs, there has been a growing number of studies focusing on the combination of chemotherapeutic drugs and chemosensitizers. Chemosensitizers are compounds that are not cytotoxic to cancer cells, but when combined with chemotherapy drugs it can enhance their effects (Karagounis et al., 2016). Autophagy inhibitors are one of the common chemosensitizers (Wang et al., 2021; Wong, 2021). When exposed to chemotherapeutic drugs, tumour cells tend to increase the level of intracellular autophagy, allowing them to evade the chemotherapeutic stimulus and develop resistance to the drugs (Li et al., 2017b; Smith and Macleod, 2019). Thus, co-delivery of chemotherapeutic agents with autophagy inhibitors may be a beneficial strategy for treating BC. Recent research has shown that the antimalarial drug hydroxychloroquine (HCQ) has an inhibitory effect on cellular autophagy and is a lysosomal inhibitor (Vyas et al., 2022). Its mechanism of action is to affect the degradation of autophagosomes in the later stages of autophagy by inhibiting the fusion of autophagosomes with lysosomes, resulting in the accumulation of autophagosomes, and therefore has potential application in synergy with chemotherapeutic drugs (Piao et al., 2017; Onorati et al., 2018; Amaravadi et al., 2019). In summary, if DOX and HCQ are simultaneously encapsulated in a NDDS, it can not only successfully reduce the high autophagy level of tumour cells caused by chemotherapeutic drugs and enhance the sensitivity of tumour cells to the drugs, but also simultaneously avoid the adverse effects of both drugs on normal tissues.

For NDDSs, the biocompatibility of the delivery system, various physicochemical properties and the ability to release precisely within the cell depend on the choice of biomaterial. According to the survey, chitosan (CS) is a biocompatible and biodegradable polymer with mucosal adhesion and high solubility under acidic conditions (Muxika et al., 2017; Ryu et al., 2020). Alginate (ALG) is a natural polysaccharide consisting of D - mannuronic acid and L - guluronic acid with excellent properties of biodegradability and low toxicity (Natrajan et al., 2015; Rastogi and Kandasubramanian, 2019). The electrostatic binding between CS and ALG increases in an acidic environment, thus allowing the nanomaterial composed of both to protect the drug from excessive release in an acidic environment (Zhang et al., 2018). Poly (D, L-lactide-co-glycolide) (PLGA) is a widely used functional polymeric organic compound, also with remarkable biocompatibility, which is widely used in the preparation of nanodelivery vehicles (Dong and Feng, 2005).

In this study, we designed and prepared Co-NDDSs (FeAC-DOX@PC-HCQ NPs) that encapsulated two drugs simultaneously, where the chemotherapeutic drug DOX was encapsulated in the smaller nanoparticle FeAC-DOX NPs, and then the autophagy inhibitor HCQ was co-encapsulated with FeAC-DOX NPs in the larger NPs. In addition, we characterised their physical and chemical properties (e.g., particle size, zeta potential, transmission electron microscopy, Fourier transform infrared spectroscopy and encapsulation efficiency) and further investigated their anti-cancer ability *in vitro* and their effect in inhibiting autophagy.

## 2 Materials and methods

### 2.1 Materials

All reagents and solvents were obtained from commercial suppliers and were used without further purification. Doxorubicin and 2,3-dimercaptosuccinic acid (DMSA) coated Fe_3_O_4_ NPs were acquired from Solarbio (Beijing, China). Hydroxychloroquine sulfate was obtained from TCI (Tokyo, Japan). PLGA (lactide: glycolide = 50:50, ester terminated, Mw = 38,000–54,000) and Polyvinylal-cohol (PVA, 86.5%–89% hydrolyzed, viscosity 4.6–5.4 mPa·s were purchased from Aladdin (Shanghai, China). Chitosan (molecular weight, 50,000–190,000; viscosity, 20–30cP; and deacetylation 75%) and sodium alginate (low viscosity, 80,000–120,000; molecular weight, viscosity 2000cP) were obtained from Sigma-Aldrich (St. Louis, MO, UnitedStates). All other materials are analytical grade. Distilled water is used throughout the process.

### 2.2 Synthesis of FITC labeled CS

The synthesis of chitosan labelled by FITC is based on the reaction between the isothiocyanate group of FITC and the primary amino group of chitosan (Moussa et al., 2013). CS was dissolved in 0.1 M acetic acid solution to give 20 mL of 1% (w/v) chitosan solution. 20 mL of FITC solution (20 mg dissolved in 20 mL of dehydrated methanol) was added to the above solution. The reaction was stirred in the dark for 4 h. The pH was adjusted to 10 with 0.5 M sodium hydroxide solution to precipitate the labelled product (FITC-CS). Centrifuge and wash with distilled water until the supernatant is free of fluorescence. The labelled product was redissolved in 20 mL of 0.1 M acetic acid solution and dialyzed in 4 L of distilled water in the dark for 3 days, with daily water changes.

### 2.3 Preparation of NPs

#### 2.3.1 Preparation of drug-loaded NPs

Based on the existing literature, DOX was wrapped around the DMSA coated Fe_3_O_4_@DMSA NPs (Oh et al., 2017). Briefly, about 0.1mLFe_3_O_4_@DMSA (∼4mg/mLFe_3_O_4_) were incubated with 0.5mL of DOX solution (500mg DOX) at 4°C for 12 h to obtain DOX coupled Fe_3_O_4_@DMSA-DOX NPs, which were washed several times with water to remove the unattached free DOX molecules. Then, add 3 mL of ALG solution under gentle stirring, ultrasonic for 10 min, centrifuge at 11,000 g for 20 min and washed off the residual ALG with water. Finally, a total of 3 mL of CS solution (1% w/v acetic acid dissolved in water) was dropped at a rate of 2 drops/s, stirred for 1 h and washed off the residual CS with water. Specifically, centrifuge three times and discard the waste liquid and re-add single distilled water after each centrifugation. The above NPs were collected and freeze-dried for later use, resulting in the smaller NPs in this study, which were named FeAC-DOX NPs.

Larger NPs were prepared by the water-in-oil-in-water (W/O/W) double-emulsion solvent diffusion-volatilization method (Jin et al., 2021). FeAC-DOX NP and 4 mg of HCQ were mixed and added to 10 mL of 2% (w/v) PVA solution as the water phase, and the water phase was added dropwise to PLGA solution (80 mg/3 mL acetone) and sonicated for 1 min to form an O/W emulsion. The emulsion was poured into 100 mL of distilled water and stirred for 3 h to evaporate the organic solvent to form PLGA NPs. A total of 20mL of CS solution (1% w/v acetic acid dissolved in water) was added dropwise at a rate of 2 drops/s, stirred for 1 h and the residual CS was washed away with water. The above NPs were collected and freeze-dried for further study, resulting in the larger NPs in this study, which were named FeAC-DOX@PC-HCQ NPs.

#### 2.3.2 Preparation of tracing NPs

FITC-labelled NPs (FeAC-DOX@PC-FITC-HCQ) were prepared on the basis of the NPs prepared above. The FITC-loaded CS were used as the outer chitosan layer and were prepared in the same way as described above.

### 2.4 Physical and chemical characterization

FeAC-DOX@PC-HCQ was observed using a transmission electron microscope (TEM, H7650; Hitachi, Tokyo, Japan) at an accelerating voltage of 100 kV. The deionised water-diluted sample suspension was placed on a copper grid coated with carbon film. After drying, the samples were observed by TEM. The size distribution of NPs and zeta potential were determined by dynamic light scattering (DLS) and electrophoretic light scattering (ELS) using Zetasizer Nano ZS (Malvern Instruments, UK). All samples were resuspended in deionised water and three measurements were required, with results expressed as mean ± standard deviation (SD). The raw material, empty NPs and drug-loaded NPs were measured by Fourier transform infrared spectroscopy (FT-IR, Alpha type, Bruker, Billerica, MA, UnitedStates) in the transmittance range of 4,000-400 cm^-1^ and the chemical structure of each sample was analysed.

### 2.5 Pharmaceutical characterization

#### 2.5.1 Encapsulation efficiency (EE) and drug loading capacity (LC)

Standard curves were obtained for the concentrations of DOX and HCQ in the aqueous phase using a UV-Vis spectrophotometer (Thermo Fisher Scientific, Wilmington, DE, UnitedStates) at 480 nm and 343 nm. The amount of DOX in FeAC-DOX NPs was determined indirectly by measuring the amount of free drug in the supernatant after three washes of FeAC-DOX NPs. After the supernatant was collected, the absorbance of the solution was measured at 343 nm by UV spectrophotometry and the amount of free drug was calculated from the standard curve.

For HCQ, the amount of free drug in the supernatant of FeAC-DOX@PC-HCQ NPs after 3 washes was determined by the same method, and the amount of HCQ in FeAC-DOX@PC-HCQ NPs was determined indirectly. The EE and LC formulas are as follows:

$$EE\%=\frac{Total\;DOX\;orHCQ\;weight\;–\;Free\;DOX\;orHCQ\;weight}{Total\;DOX\;or\;HCQ\;weight}\times 100\%$$

$$LC\%=\frac{Total\;DOX\;or\;HCQ\;weight-Free\;DOX\;or\;HCQ\;weight}{Total\;NPs\;weight}\times 100\%$$

#### 2.5.2 *In Vitro* drug release

The *in vitro* release characteristics of FeAC-DOX@PC-HCQ NPs and FeAC-DOX NPs were studied by the classical dialysis bag method for *in vitro* release studies. For FeAC-DOX@PC-HCQ NPs, approximately 5 mg of FeAC-DOX@PC-HCQ NPs were suspended in 5 mL of buffer solution at different pH values (5.8 and 7.4) to simulate the cytoplasmic (pH 5.8) and physiological (pH 7.4) environments of cancer cells. The suspension was then transferred to a dialysis bag (molecular weight 7 K, Solarbio, Beijing, China), placed in 200 mL of the same pH PBS and incubated at 37°C at 70 rpm. 2 mL of HCQ release solution was removed at predetermined time intervals and the HCQ concentration was measured at 343 nm using a UV spectrophotometer while an equal amount of fresh buffer was added.

For FeAC-DOX NPs, NPs were suspended in 5 mL of buffer solution at different pH values (5.0, 5.8 and 7.4) to simulate lysosomes (pH 5.0), the cytoplasmic environment of cancer cells (pH 5.8) and the nucleus (pH 7.4). DOX concentrations were measured at 480 nm by the above method. The cumulative drug release formula is as follows:

$$The\;cumulative\;drug\;release\%=\frac{HCQ\;or\;DOX\;released\;amount}{total\;amount\;of\;HCQ\;or\;DOX\;in\;NPs}\times 100\%$$

### 2.6 *In Vitro* biological effect experiment

#### 2.6.1 Cell culture

MCF-7 and MDA-MB-231 human BC cells were cultured at 37°C in a humid atmosphere containing 5% CO_2_ (Thermo Fisher Scientific), and the cells were cultured in DMEM medium. Penicillin (100 U/mL), streptomycin (100 U/mL) and heat-inactivated fetal bovine serum (10%, Atlanta Biologics; Flowery Branch, GA, UnitedStates) were added to the medium.

#### 2.6.2 *In Vitro* cytotoxicity of empty NPs and drug-loaded NPs

In the cytotoxicity assay with material and drug-loaded NPs, cell concentrations were first determined by hemocytometry, cells were inoculated in 96-well plates at a density of 5 × 10^3^ cells per well and incubated at 37°C in a 5% CO_2_ atmosphere for 24 h. The original medium was removed and MCF-7 and MDA-MB-231cells were treated with 100 μL of medium containing DOX at concentrations ranging from 0 to 10.0 μg/L (actual DOX content was calculated based on the loading of NP) in different concentrations of empty NP, free DOX, free DOX + HCQ, FeAC-DOX NPs and FeAC-DOX@PC-HCQ NPs, and set blank and control groups. The concentration of HCQ in this co-administration system was a fixed value and 10 μg/mL low cytotoxicity was chosen for the experiment. At the fixation time (24 h), the medium containing the material and drug-loaded NPs was removed and the cells were washed twice with PBS. Then 10 μL of CCK-8 reagent was added and the plates were placed in an incubator for 1.5 h. Finally, absorbance was measured at 450 nm using a microplate reader. A treatment concentration of 50% inhibition (IC 50) was used for further studies. Each set of experiments was repeated three times. The formula for cell viability was as follows:

$$The\;cell\;viability=\frac{OD\;treat-OD\;blank}{OD\;control-OD\;blank}\times 100$$

#### 2.6.3 *In Vitro* migration assay

The effect of different drugs on the migration of BC cells was also assessed by a wound healing assay. MCF-7 and MDA-MB-231 cells were pre-inoculated in 6-well plates and cell monolayers were carefully scratched with the tip of a 10 μL sterile pipette when the cells had grown to a 90% concentration. Cell debris was washed with PBS and photographed under a microscope as a control at 0 h. After incubation with different drugs for 48h, photographs were taken for comparison with the 0 h control and the relative migration area of the cells was calculated using ImageJ.

#### 2.6.4 Cellular uptake

Experiments were performed using FITC-labelled NPs (FeAC-DOX@PC-FITC-HCQ NPs) and cellular uptake of NPs was analysed by fluorescence microscopy imaging. Briefly, MCF-7 and MDA-MB-231 cells were inoculated in 24-well plates and incubated in a 5% CO_2_ incubator at 37°C for wall attachment. Drug-containing medium was then added and the cells were incubated together for 0 h, 6 h, 12 h and 24 h. The medium was aspirated and excess NPs was removed using PBS. The cells were then fixed with 4% paraformaldehyde for 20 min and DAPI was added for staining for 5 min. The images were viewed with an Olympus fluorescence microscope, and the images were acquired using the RBITC channel, DAPI channel and DOX intrinsic red fluorescence.

### 2.7 Autophagy analysis

#### 2.7.1 Western blot assay

The expression of autophagy-associated marker protein (LC3) was detected by immunoblotting. MCF-7 and MDA-MB-231 cells from different groups were collected after 24 h incubation with drugs, and approximately 100 mL of lysate was added to each group separately to ensure that all proteins were solubilized. The proteins extracted from each group of cells were collected and they were diluted to the same concentration. Each group of proteins was electrophoresed with equal micrograms of SDS-PAGE and then transferred onto polyvinylidene fluoride membranes (PVDF). After blocking for 60 min, the monoclonal antibodies LC3 (1:1000, Affinity) and β-actin (1:100,000, Abclonal) were incubated overnight at 4 °C respectively. After incubation, protein expression is observed after incubation with goat anti-rabbit HRP antibody (1: 8,000, Abclonal) for 60 min at room temperature. The assay was repeated at least three times. Bands were quantified using ImageJ.

#### 2.7.2 Monodansylcadaverine (MDC) staining assay

MDC staining was performed to detect autophagic vesicles in MCF-7 and MDA-MB-231 cells after different drug treatments to reflect the level of autophagy. After 24 h of treatment of cells with different drugs, cells were collected and incubated with MDC (Solarbio, Beijing, China) for 20 min at 37°C in the dark. Cells were centrifuged at 800 *g* for 5 min, washed with 1× WASH buffer and then resuspended. Cell suspensions were dropped onto slides and sealed, and staining results were observed using a fluorescent microscope (Ti2-A, Nikon, Japan).

## 3 Results

### 3.1 Preparation of NPs

**FIGURE 1:**
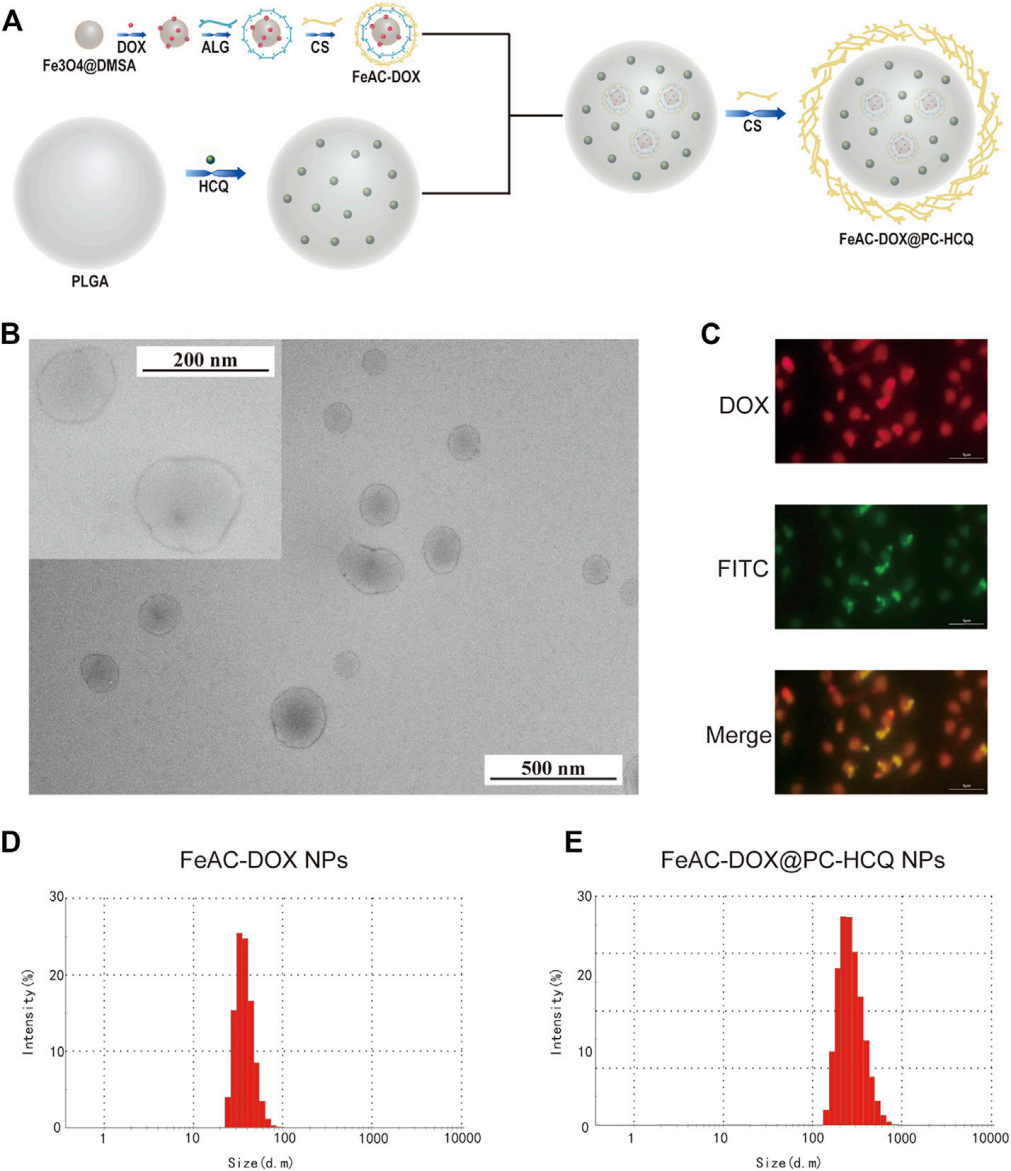
Preparation and characterization of co-delivery NPs. **(A)** Preparation scheme of co-delivery NPs. **(B)** TEM images of co-delivery NPs under 200 nm field of view and 500 nm field of view. **(C)** The fluorescence images of FeAC-DOX@PC-FITC-HCQ NPs. The average particle size of FeAC-DOX NPs **(D)** and FeAC-DOX@PC-HCQ NPs **(E)** detected by Zetasizer Nano ZS.

Firstly, smaller NPs loaded with DOX (FeAC-DOX) were prepared using the ionic gelation method by exploiting the electrostatic interactions between the self-carboxyl groups of Fe_3_O_4_@DMSO nanomaterials and the amino groups of DOX, and between the negatively charged carboxyl groups in ALG and the positively charged amino groups in CS. Next, FeAC-DOX NPs and HCQ were used as the aqueous phase and PVA was used as the emulsifier to encapsulate them in PLGA using the O/W emulsion technology, and CS was added to the outermost layer to make the NPs more stable as a whole. This procedure resulted in the preparation of larger NPs loaded with both DOX and HCQ (FeAC-DOX@PC-HCQ NPs), as shown in Figure 1A.

### 3.2 Physical and chemical characterization

Observation of FeAC-DOX@PC-HCQ NPs under transmission electron microscopy shows that FeAC-DOX@PC-HCQ NPs loaded with both FeAC-DOX NPs and HCQ are approximately spherical and have a smooth surface with a diameter of about 200 nm. Figure 1B shows the field of view of FeAC-DOX@PC-HCQ NPs at 200 nm and 500 nm.

The fluorescence tracking technique was used to verify that the smaller NPs FeAC-DOX NPs is co-wrapped with HCQ in the larger NPs FeAC-DOX@PC-HCQ NPs (Figure 1C). Since HCQ does not have fluorescent properties while DOX is self-fluorescent in red, FITC-labelled NPs (FeAC-DOX@PC-FITC-HCQ NPs) were prepared to trap HCQ by using the FI-TC-loaded CS as the outermost layer of the NPs. In the fluorescence images, the outer CS and DOX exhibited green and red fluorescence, respectively. The results show that the yellow fluorescence appears in the centre of the NPs, indicating the superposition of red (DOX) and green (HCQ) fluorescence, which indicates that FeAC-DOX NPs are co-encapsulated with HCQ in FeAC-DOX@PC-HCQ NPs.

The specific size distribution of the above NPs as well as the potential were measured by dynamic light scattering. FeAC-DOX NPs is classified as 59.3 ± 7.1 nm **(** Figure 1D) and FeAC-DOX@PC-HCQ NPs as 255.7 ± 18.45 nm (Figure 1E) with PDI values of 0.337 ± 0.101 and 0.224 ± 0.083 respectively (Table 1), similar to those observed under transmission electron microscopy, with suitable dimensions and good dispersion. Among the many parameters, the size of the NPs played an important role in the EPR effect, with a cut-off size of approximately 400 nm for penetration into the tumour, with particles less than 200 nm in diameter being the most effective (Sun et al., 2014). The Zeta potential of FeAC-DOX@PC-HCQ NPs was +22.3 ± 0.8 mV and that of FeAC-DOX NPs. The uptake of the negatively charged nanodrugs by tumour cells was increased, and the ability to escape from lysosomes after cell entry was also improved, resulting in enhanced anticancer efficacy (Du et al., 2011).

**TABLE 1:** Mean diameter and zeta potential of nanoparticles.

Nanoparticles	Size (nm)	PDI	Zate potential (mV)
FeAC-DOX NPs	59.3 ± 7.1	0.337 ± 0.101	+13.6 ± 2.1
FeAC-DOX@PC-HCQ NPs	255.7 ± 18.45	0.224 ± 0.083	+22.3 ± 0.8

**FIGURE 2:**
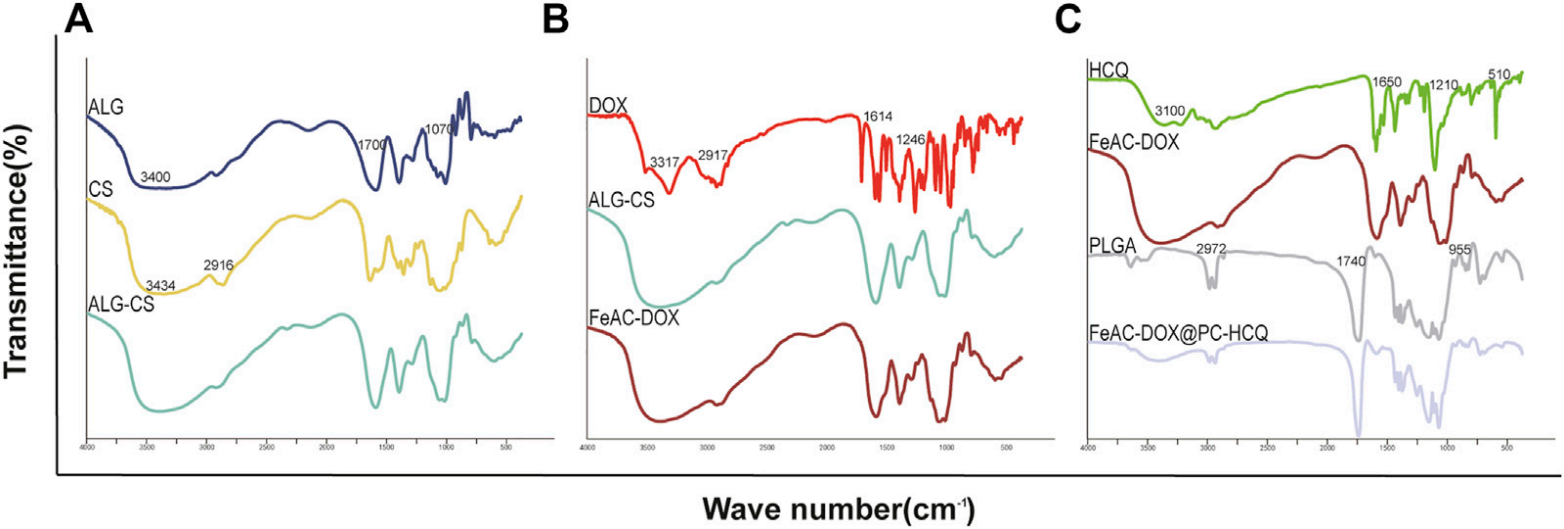
The chemical structure of the raw materials, empty NPs, and drug-loaded NPs are analyzed by FT-IR. **(A)** ALG, CS, ALG-CS NPs; **(B)** DOX, ALG-CS NPs, FeAC-DOX NPs; **(C)** HCQ, FeAC-DOX NPs, PLGA, FeAC-DOX@PC-HCQ NPs.

DOX, HCQ, ALG, CS, PLGA, ALG-CS NPs, FeAC-DOX NPs and FeAC-DOX@PC-HCQ NPs were chemically characterised by FT-IR spectroscopy, and Figure 2 shows the potential interactions between them. The basic characteristic peaks for ALG appear at 3400 cm^-1^ (O-H stretching and N-H stretching, overlapping), 1700 cm^-1^ (C=O stretching), 1070 cm^-1^ (C-O-C stretching) and 955 cm^-1^ (O-H stretching). The IR spectrum of CS shows characteristic peaks at 3434 cm^-1^ (O-H stretching and N-H stretching, overlapping), 1596 cm^-1^ (N-H stretching), 1465 cm^-1^ (C-H stretching) and 1150 cm^-1^ (C-O-C stretching). The infrared spectra of CS-ALG NPs demonstrate a stretching vibration shift of -OH and -NH_2_ at 3434 cm^-1^ to 3424 cm^-1^ and a stretching shift of the -COO- group to 1579 cm^-1^. The results demonstrate that the amino group of CS reacts with the carboxyl group of ALG to form an amide group and these changes are chemically bound.

The basic characteristic peaks of DOX appear at 3317 cm^-1^ (O-H stretching), 2917 cm^-1^ (C-H stretching), 1729 cm^-1^ (C=O stretching), 1614 cm^-1^ (C=C in the benzene ring) and 1246 cm^-1^ (C-O stretching). The disappearance of the characteristic absorption peaks of DOX in FeAC-DOX NPs may indicate that DOX has been successfully encapsulated in FeAC-DOX NPs.

In addition, the HCQ results show characteristic peaks for N-H at 3319 cm^-1^ and 3100 cm^-1^ and three peaks at 1650 cm^-1^, 1571 cm^-1^ and 1501 cm^-1^ suggesting the presence of an aromatic ring structure. C-H in PLGA has a broadband at 2972 cm^-1^, C=O has an absorption peak at 1740 cm^-1^, C-H produces absorption peaks at 1470 cm^-1^ and 1380 cm^-1^ stretching, and O-H produces an absorption peak at 955 cm^-1^ from carboxyl vibrations. After the formation of FeAC-DOX@PC-HCQ NPs, some of the absorption peaks weaken or disappear in intensity and the wave number shifts. For example, the IR spectrum of the final NPs shows a weakening of the broad absorption peak at 2972 cm^-1^ and the appearance of the broad absorption peak at 1650 cm^-1^. These changes suggest that the formation of FeAC-DOX@PC-HCQ NPs is not a physical combination of the various materials and drugs, but a chemical reaction between them, meaning that both DOX and HCQ drugs are successfully encapsulated in the final NPs.

The drug EE and LE of FeAC-DOX NPs and FeAC-DOX@PC-HCQ NPs were 81.7% and 67.2% and 6.71% and 8.94%, respectively (Table 2). These results suggest that the co-delivery NPs are ideal NDDSs.

**TABLE 2:** Encapsulation efficiency (EE) and loading efficiency (LE) of nanoparticles.

Nanoparticles	EE of DOX (%)	LC of DOX (%)	EE of HCQ (%)	LC of HCQ (%)
FeAC-DOX NPs	81.7	6.71	-	-
FeAC-DOX@PC-HCQ NPs	-	-	67.2	8.94

### 3.3 *In Vitro* drug release

**FIGURE 3:**
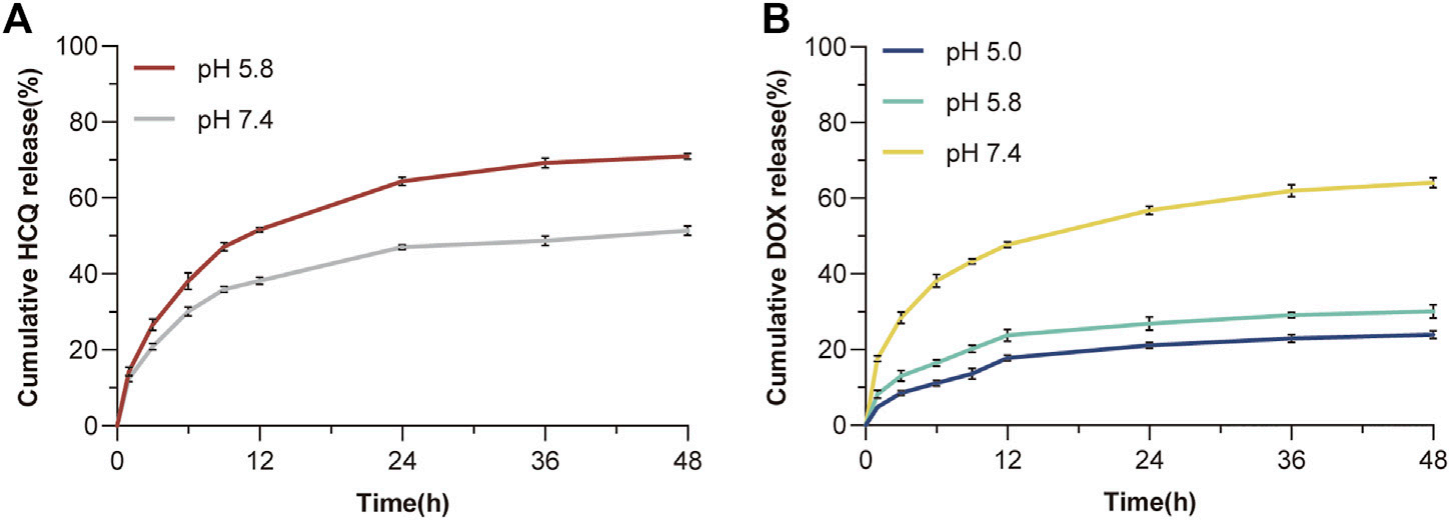
*In vitro* drug release. **(A)** The release curve of HCQ from FeAC-DOX@PC-HCQ NPs in PBS (pH 5.8 and pH 7.4). **(B)** The release curve of DOX from FeAC-DOX NPs in PBS (pH 5.0, pH 5.8, and pH 7.4).

The ability of the synthesised larger NPs FeAC-DOX@PC-HCQ NPs to release the drug in buffers of different pH was examined by a classical cumulative drug release method to simulate a neutral blood environment or an acidic tumour cell environment. Figure 3A shows the cumulative release of HCQ from larger NPs (FeAC-DOX@PC-HCQ NPs) at pH values of cancer cell cytoplasm (pH 5.8) and blood or normal organs (pH 7.4). The cumulative release of FeAC-DOX@PC-HCQ NPs at pH 5.8 was 71.1% at 48 h and the cumulative release of HCQ at pH 7.4 was 51.3% at 48 h, respectively, indicating that the drug release from the NPs in the simulated blood or normal organ environment was less than that in the simulated cancer cell cytoplasm. For the smaller nanoparticle FeAC-DOX NPs, their ability to release DOX was assayed by the same method (Figure 3B), including simulated lysosomal environment (pH 5.0), cancer cell cytoplasmic environment (pH 5.8) and cytosolic environment (pH 7.4). The results showed that the cumulative DOX release from FeAC-DOX NPs at pH 7.4 was 64.1% for 48 h, while at pH 5.8 and pH 5.0, the cumulative DOX release for 48 h was 30.1% and 24.0%, respectively.

The above results indicate that FeAC-DOX@PC-HCQ NPs is pH sensitive and that the encapsulated drug is released sequentially. From the release characteristics of the larger NPs, it can be concluded that the release of HCQ in the simulated blood environment is much smaller than that in the simulated cancer cell cytoplasm. This is due to the fact that the outer chitosan layer keeps the larger NPs relatively stable in a neutral blood environment (Li et al., 2018), whereas in the acidic environment of cancer cell cytoplasm the CS dissolves due to the weakened inter-chain interactions caused by the protonation of amino groups in the CS, resulting in the release of the drug from the NPs (Rizeq et al., 2019). For smaller NPs, FeAC-DOX NPs is more stable and less likely to release drugs in an acidic cytoplasmic environment and lysosomal environment than in a neutral environment at pH 7.4 (which can also as the pH of the nucleus). This is due to the enhanced binding of electrostatic interactions between CS and ALG in the acidic environment (Biswas et al., 2015), which slows drug release.

It is clear that FeAC-DOX@PC-HCQ NPs protect the drug encapsulated in them well during blood transport, while HCQ and smaller NPs are better released when transported into the acidic tumour cell environment, where the released HCQ will act on its pharmacological target to inhibit autophagy and protect FeAC-DOX NPs from being removed by autophagy. After lysosomal escape, the FeAC-DOX NPs protected DOX in an acidic environment (cytoplasm and lysosomes), while entering the nucleus, they release DOX at pH 7.4 and thus have a cytotoxic effect on DNA.

### 3.4 *In Vitro* biological effect

#### 3.4.1 Cell viability of empty NPs

**FIGURE 4:**
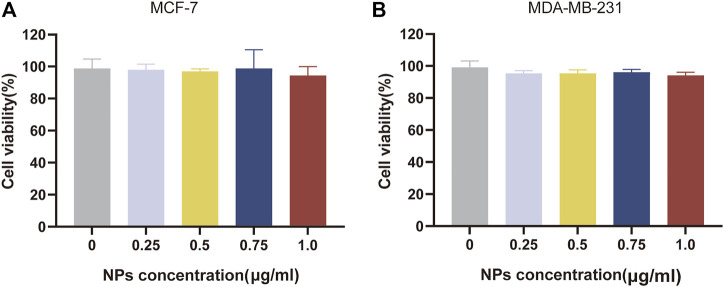
Biocompatibility evaluation of nanomaterials. *In vitro* cell viability of empty NPs at various concentrations for 24 h, respectively on MCF-7 cell **(A)** and MDA-MB-231 cell **(B)**.

Biocompatibility is a major factor in the evaluation of drug delivery systems targeting tumours. Different concentrations of blank NPs were applied to MCF-7 and MDA-MB-231 BC cells and cytotoxicity tests were performed using the CCK-8 kit to obtain cell viability. As shown in Figure 4, the concentrations of blank NPs were selected as 0.25, 0.5, 0.75 and 1.0 μg/mL. After 24 h of nanomaterial action, the different concentrations of blank NPs were not significantly toxic to MCF-7 and MDA-MB-231 BC cells, and the cell survival rates were above 95%. In conclusion, the nanomaterials selected for the study have proven to be safe as drug carriers and possess high biocompatibility.

#### 3.4.2 Cytotoxicity of drug-loaded NPs

**FIGURE 5:**
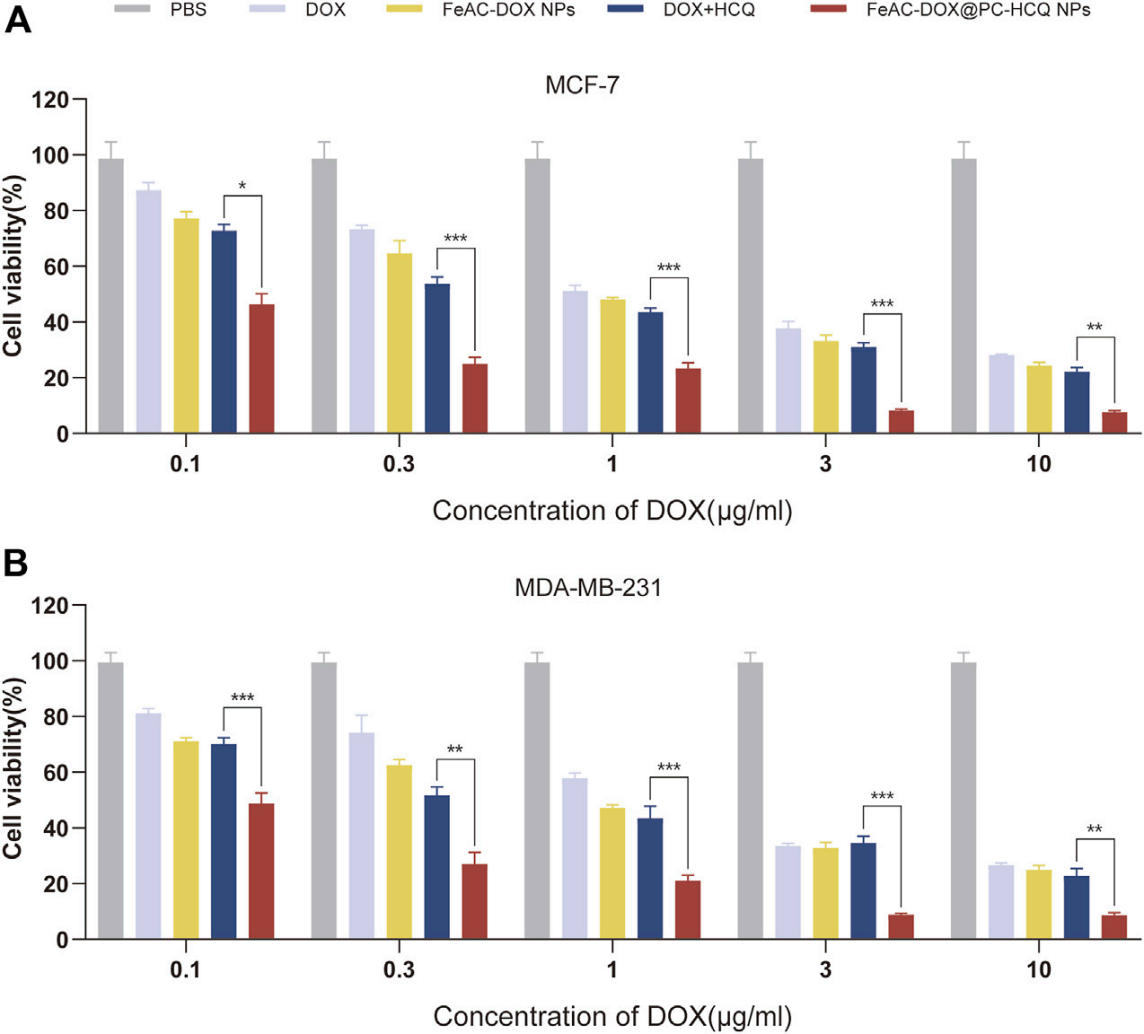
Cytotoxicity of drug-loaded NPs. The compare of *in vitro* cytotoxic between free drugs (DOX, DOX and HCQ) and drugs-loaded NPs (FeAC-DOX NPs, FeAC-DOX@PC-HCQ NPs) at various concentrations for 24 h, respectively on MCF-7 cell **(A)** and MDA-MB-231 cell **(B)**. Data represent as mean ± SD (n = 3). *p < 0.05, **p < 0.01 and ***p < 0.001.

The results of the drug cytotoxicity assay are shown in Figure 5. The cytotoxic effects of different administration forms of the model drug (free DOX, FeAC-DOX NPs, free DOX + HCQ and FeAC-DOX@PC-HCQ NPs) on MCF-7 and MDA-MB-231 cells were examined at different DOX concentrations. The results showed that NPs containing different concentrations of DOX (FeAC-DOX NPs) had a higher cytotoxic effect on both BC cells than the free DOX group. Nanopreparations containing lower concentrations of DOX (0.1–1.0 μg/mL) had significantly higher cytotoxic effects on BC cells than the free drug (*p* < 0.05), while nanopreparations containing higher concentrations of DOX (10 μg/mL) had comparable cytotoxic effects to the free DOX group. The cytotoxic effects of nanopreparations with almost all concentrations of DOX (0.1–10 μg/mL) were significantly higher than those of the free drug group for both BC cells (*p* < 0.05). Notably, the combination drug group at different concentrations (DOX + HCQ) was more potent in killing both BC cells than the chemotherapeutic drug DOX alone, and this aspect was more pronounced for MCF-7 cells.

In addition, the IC50 values of different free drug and drug-loaded NPs were compared for MCF-7 cells and MDA-MB-231 cells. From the experimental results (Table 3), it can be seen that the nanoformulation of DOX (FeAC-DOX NPs) showed lower IC50 values than free DOX in both cell lines. The NDDS increased the killing effect of DOX on MCF-7 and MDA-MB-231 cells by 25.80% and 23.39%, respectively. For the co-administration of DOX and HCQ, the cytotoxic effect of co-loaded nano-x on both BC cells also reached 2 and 4.54 times that of the two free drugs, respectively, and this killing effect was even more pronounced for MDA-MB-231 cells.

**TABLE 3:** IC50 values of different formulations at 24 h.

Formulation	IC50(μg/mL)	
	MCF-7	MDA-MB-231
DOX	0.62	1.24
FeAC-DOX NPs	0.46	0.95
DOX + HCQ	0.32	0.59
FeAC-DOX@PC-HCQ NPs	0.16	0.13

#### 3.4.3 Effect of drug-loaded NPs on cell migration

**FIGURE 6:**
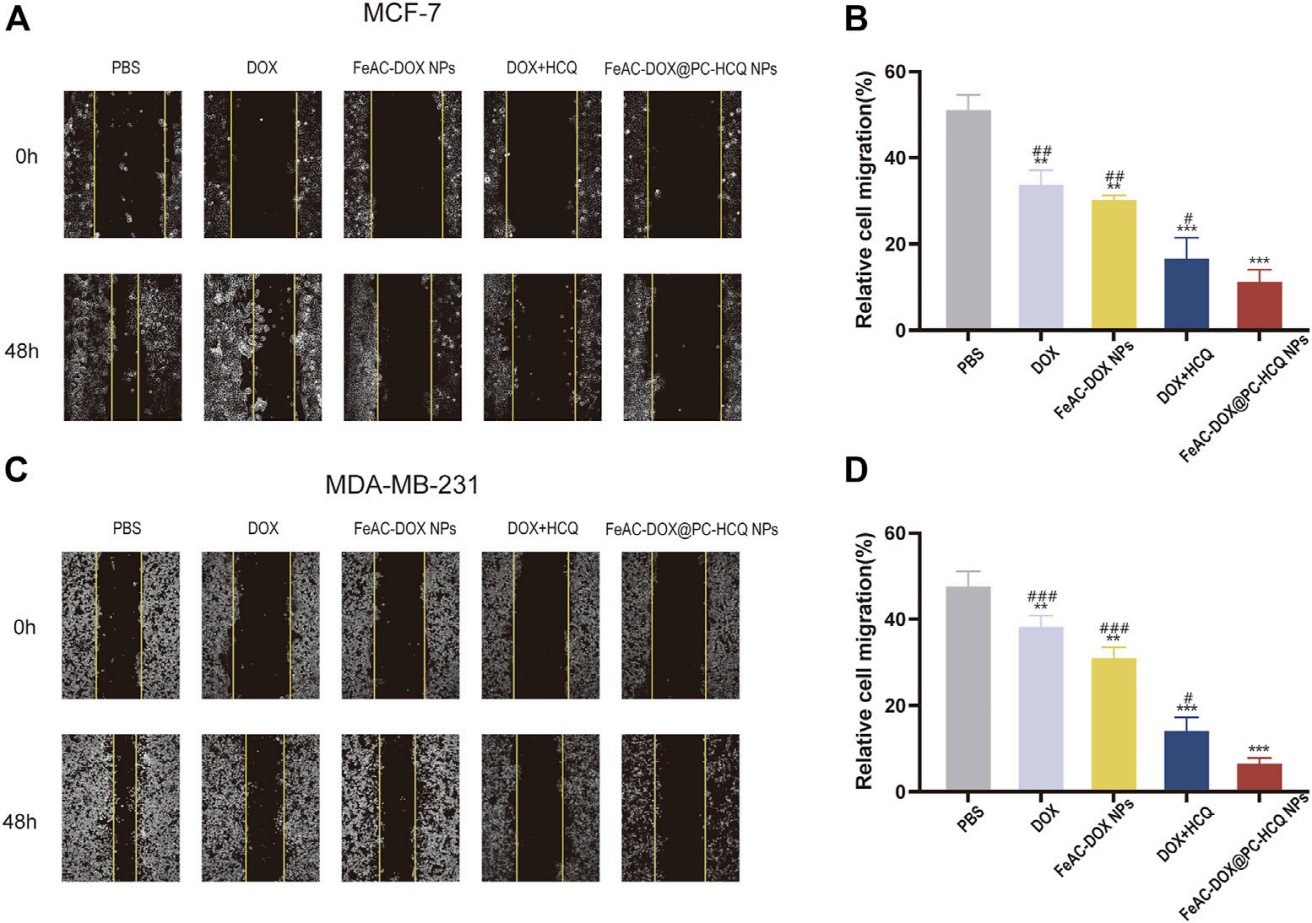
Effect of drug-loaded NPs on cell migration. Representative images and relative cell migration treated with PBS, free drugs (DOX, DOX and HCQ) and drugs-loaded NPs (FeAC-DOX NPs, FeAC-DOX@PC-HCQ NPs) for 48 h, respectively on MCF-7 cell **(A, B)** and MDA-MB-231 cell **(C, D)**. Data represent as mean ± SD (*n* = 3). *p < 0.05, **p < 0.01 and ***p < 0.001. *vs.* PBS. #*p* < 0.05, ##*p* < 0.01 and ###*p* < 0.001. *vs* FeAC-DOX@PC-HCQ NPs.

The migration of tumour cells is closely related to tumour metastasis, which is a major factor contributing to high mortality (Ko et al., 2021). Figures 6A, C demonstrate the effects of different model drugs on the migration of MCF-7 cells and MDA-MB-231 cells *in vitro*, respectively. Compared to the control group, each drug treatment group inhibited the migration of BC cells to different degrees (*p* < 0.01 or *p* < 0.001), with the most pronounced inhibition occurring in the co-loaded NPs (FeAC-DOX@PC-HCQ NPs) treated group. In the cell migration assay, control cells gradually migrated towards the nutrient-rich and low survival pressure scratches, which recovered significantly after 48 h incubation. For MCF-7 cells, cell migration rates were 34.1%, 30.6%, 17.1% and 11.7% after 48 h incubation by medium containing the same drug loading of free DOX, DOX nanoformulation (FeAC-DOX NPs), free DOX + HCQ, DOX and HCQ co-loaded nanoformulation (FeAC-DOX@PC-HCQ NPs), respectively. In contrast, for MDA-MB-231 cells, the cell migration rates after the same treatment were 38.6%, 31.4%, 14.5% and 6.9%, respectively (Figures 6B, D). The results indicated that the nanoformulation enabled the drugs to inhibit BC cell migration more significantly than the free drugs, while the combined drug group was more able to inhibit tumour cell migration than the group treated with chemotherapeutic drugs alone.

#### 3.4.4 Cellular uptake of NPs

**FIGURE 7:**
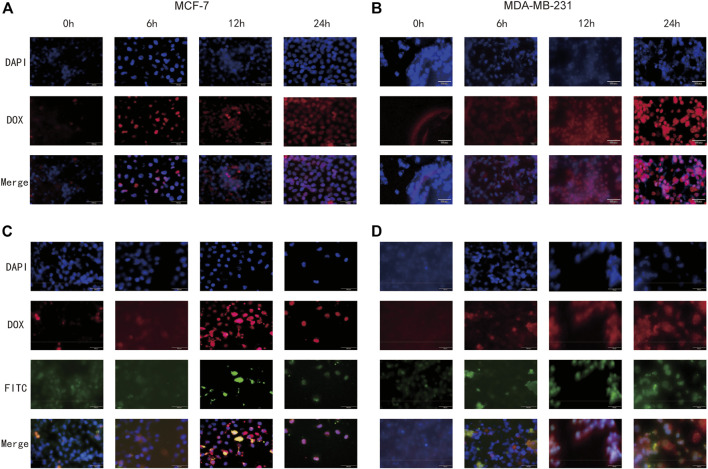
Cellular uptake of free DOX and FeAC-DOX@PC-FITC-HCQ NPs by MCF-7 cells and MDA-MB-231 cells, respectively. MCF-7 cells **(A)** and MDA-MB-231 cells **(B)** were treated with free DOX (red) for 0, 6, 12 and 24 h, and the fixed cells were stained with DAPI to observe the nucleus (blue). MCF-7 cells **(C)** and MDA-MB-231 cells **(D)** were treated with FeAC-DOX@PC-FITC-HCQ NPs for 0, 6, 12 and 24 h. Red fluorescence indicates DOX loaded in NPs, green fluorescence indicates FITC loaded in NPs, and blue fluorescence indicates the region of the nucleus stained with DAPI.

Whether NPs can be effectively taken up by cells is a major requirement for evaluating their therapeutic efficacy. To investigate the detailed uptake of drugs in cancer cells, free DOX and FeAC-DOX@PC-HCQ NPs were co-incubated with MCF-7 and MDA-MB-231 BC cells for 0 h, 6 h, 12 h and 24 h, respectively (Figure 7).

Figures 7A, B show the uptake of free DOX by both cell lines, with blue fluorescence representing the DAPI-stained nucleus, red fluorescence representing DOX and purple fluorescence representing the overlapping area between the two. As the results show, when the cells were treated with free DOX for 0 h, no overlap between blue and red fluorescence was seen, indicating that no free DOX was entering the cells at this time. After 6 h of co-incubation, red fluorescence appeared near the nucleus, and as the time was extended to 12 h, the blue fluorescence and red fluorescence came further together and even partially overlapped. At 24 h, the overlapping purple fluorescence continued to increase, indicating that the free drug had effectively entered the nucleus at this time, and this phenomenon was more fully expressed in MCF-7 cells.

The internalization process and intracellular localisation of the FeAC-DOX@PC-HCQ nanoparticle drug in two BC cell lines was then proceeded to be examined using fluorescent tracer techniques (Figures 7C, D). Since HCQ is loaded in larger NPs without fluorescent properties, FeAC-DOX@PC-FITC-HCQ NPs were prepared for fluorescent tracing for this purpose. In this fluorescent tracer system, DOX and FITC exhibit red and green fluorescence, respectively, and blue fluorescence remains representative of the DAPI-stained nuclei. Thus, under fluorescence microscopy, the yellow fluorescence is an overlap of red (DOX) and green (FITC) fluorescence, and the purple fluorescence represents a fusion of blue (DAPI) and red (DOX) fluorescence. From 0 h to 12 h, yellow fluorescence gradually accumulated around the nucleus as the incubation time increased, demonstrating that the co-administered NDDS had been effectively delivered to the pericellular area. For the cellular uptake of DOX, from 0 h to 6 h, the purple fluorescence around the nucleus gradually increased in density as the incubation time increased, demonstrating the presence of DOX in the nucleus, while from 12 h to 24 h, the purple fluorescence was clearly visible in the nucleus and continued to increase in density, indicating that DOX had accumulated in the nucleus of tumour cells and exerted anti-cancer effects. This phenomenon was also evident in both BC cell lines. This demonstrates that the NDDS can deliver the drug to the nucleus more efficiently than the free DOX, thus exerting anti-cancer effects.

### 3.5 Autophagy analysis

#### 3.5.1 LC3-II and LC3-II/LC3-I protein expression assay

**FIGURE 8:**
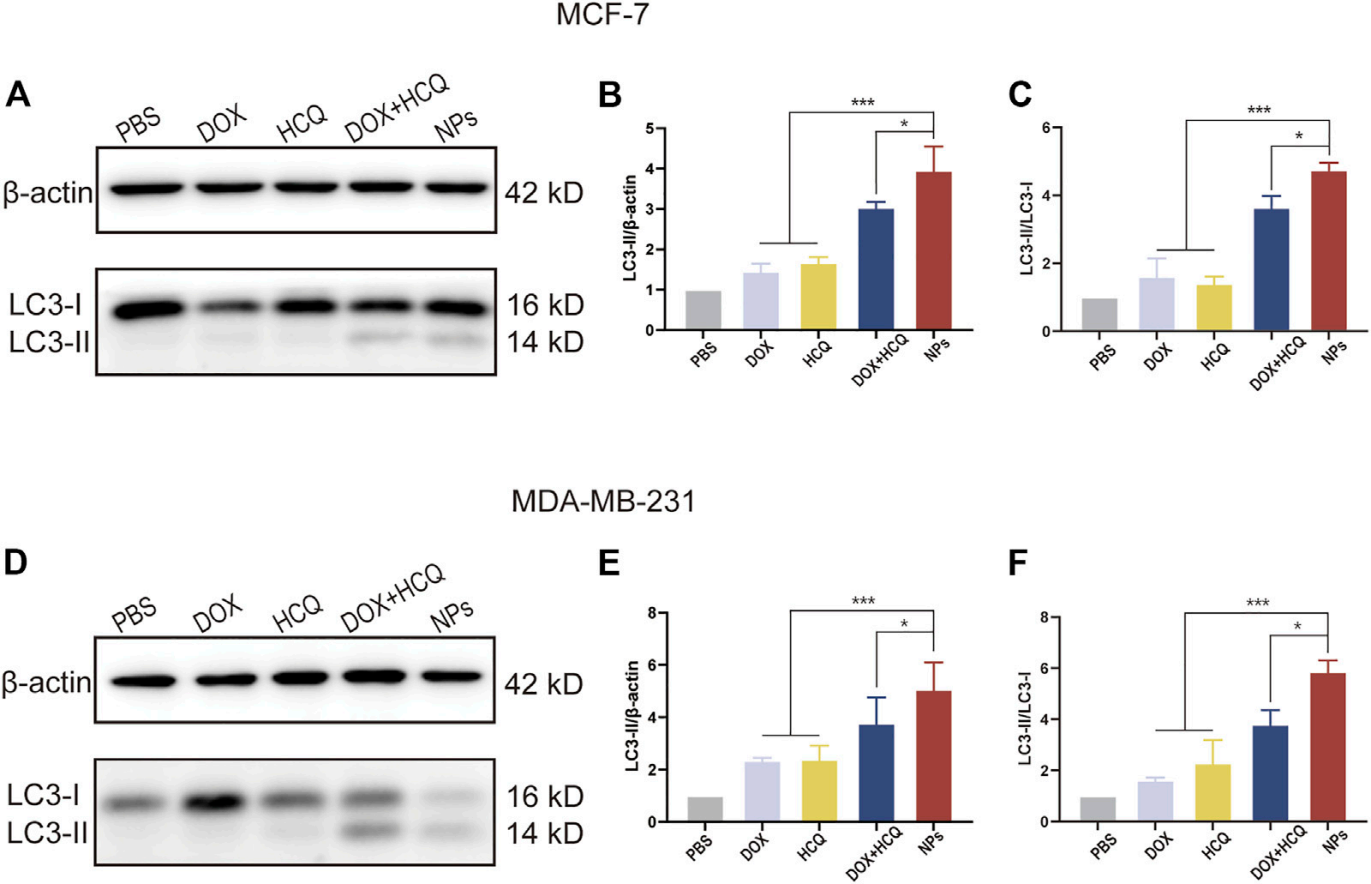
WB analysis of cellular autophagy inhibition levels in different treatment groups. The expression level of LC3-I and LC3-II, and the ratio of LC3-II/β-actin and LC3-II/LC3-I treated with PBS, free drugs (DOX, DOX and HCQ) and drugs-loaded NPs (FeAC-DOX NPs, FeAC-DOX@PC-HCQ NPs) for 24 h and their quantitative analysis, respectively on MCF-7 cell **(A–C)** and MDA-MB-231 cell **(D–F)**. Protein levels are normalized to βactin. Data represent as mean ± SD (*n* = 3). *p < 0.05, **p < 0.01 and ***p < 0.001.

It is well known that autophagy is strongly linked to the conversion of LC3-I proteins to autophagosome-associated LC3-II proteins in the cytoplasm, and that an increase in the LC3-II to LC3-I ratio indicates inhibition of autophagy (Song et al., 2019). To evaluate autophagy inhibition, western blotting was utilized to detect the level of LC3, an essential molecule of autophagy. Figure 8 shows the protein expression of LC3-I and LC3-II and interpretation of LC3-II/β-actin and LC3-II/LC3-I after 24 h treatment of MCF-7 and MDA-MB-231 cells with different groups of model drugs. For both BC cell lines, western blot results showed that the LC3-II and LC3-II/LC3-Iwas significantly increased in the DOX and HCQ combination administration group compared to free DOX or free HCQ alone (*p* < 0.001). The chemotherapeutic drug DOX induced autophagy, while HCQ prevented the fusion of endosomes with lysosomes, leading to the accumulation of LC3-II and thus the cessation of autophagy at a later stage. In contrast, the LC3-II and LC3-II/LC3-I was significantly higher in the co-administered nanodrug group (FeAC-DOX@PC-HCQ NPs) than in all other groups (*p* < 0.05), indicating that the accumulation of LC3-II was the highest in all groups, thus demonstrating that co-administered NPs inhibited autophagy best.

#### 3.5.2 Autophagic vesicles assay

**FIGURE 9:**
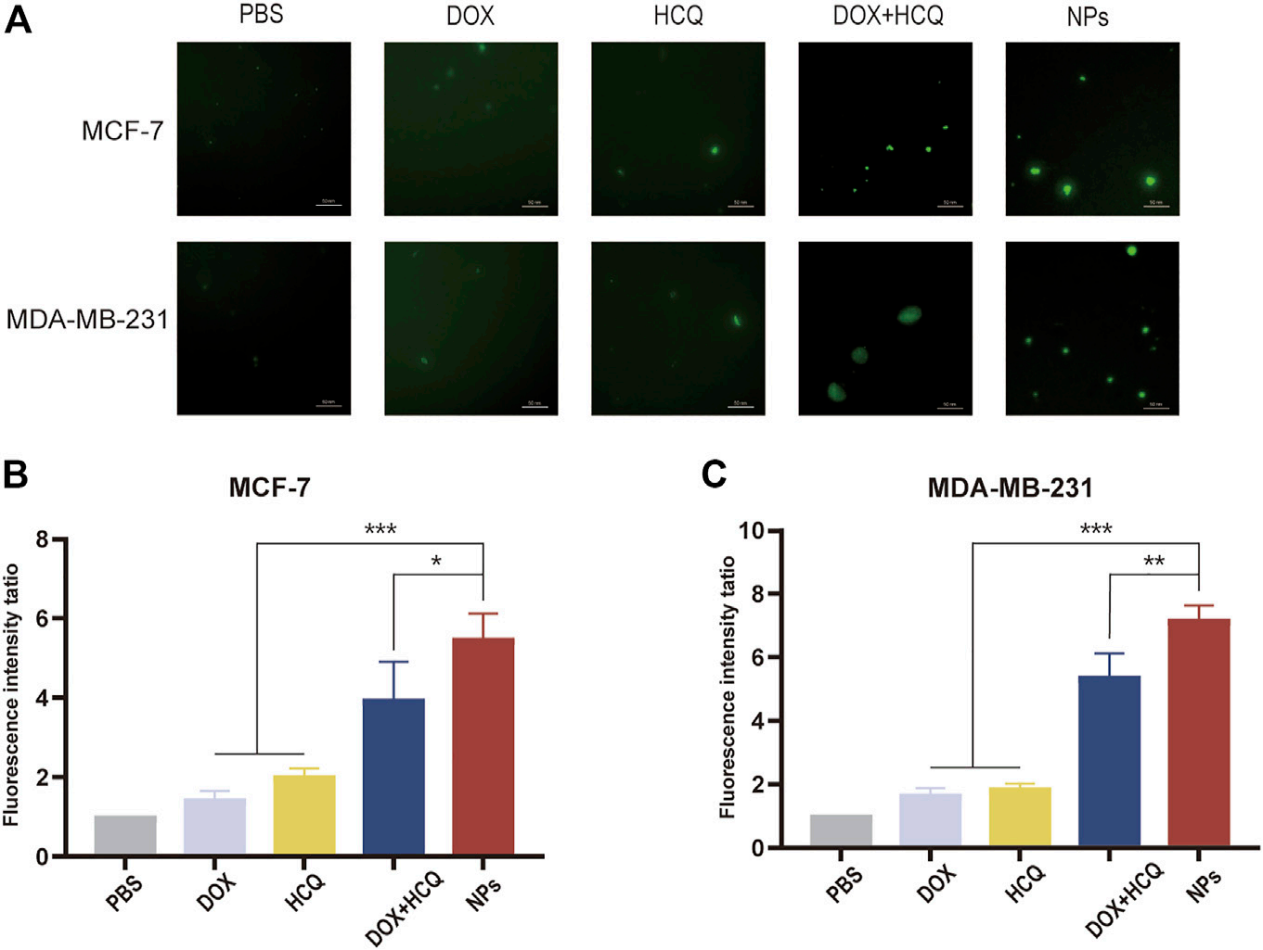
Autophagic vacuoles staining by MDC. **(A)** Fluorescence microscopy images of MDC staining of PBS, free drug (DOX, DOX, HCQ) and drug-loaded NPs (FeAC-DOX NPs, FeAC-DOX@PC-HCQ NPs) after 24 h treatment on MCF-7 cells and MDA-MB-231 cells, respectively. Quantification of MDC staining by fluorescent intensity analysis, respectively on MCF-7 cell **(B)** and MDA-MB-231 cell **(C)**. Data represent as mean ± SD (n = 3). *p < 0.05, **p < 0.01 and ***p < 0.001.

After a series of experiments to demonstrate the effectiveness of the NDDS against BC *in vitro*, to further demonstrate whether the NDDS in this experiment achieved better efficacy due to autophagy inhibition, an MDC kit was used to stain autophagic vesicles. The autophagy inhibition was observed by comparing the amount of autophagic vesicles in the cells after treatment with the model drugs in each group. The results showed that after treatment with free DOX alone, autophagic vesicles continued to increase in two different BC cell lines, indicating that the cells had started to produce autophagosomes at this point, a situation that was a hindrance to better tumour killing. In contrast, the fluorescence intensity increased significantly after DOX combined with HCQ treatment, indicating that autophagy inhibition was occurring at this point and that the destroyed organelles within the cells could not be cleared, thus improving drug efficacy. Furthermore, the intracellular fluorescence intensity after treatment with co-administered nanodrugs (FeAC-DOX@PC-HCQ NPs) was significantly higher (*p* < 0.05) than in all other groups, as demonstrated in both BC cell lines. Taken together, this further confirms that the nano-loaded system in this experiment can enhance tumour killing through autophagy inhibition Figure 9.

## 4 Discussion

In the treatment of BC, traditional chemotherapy has been gradually replaced by combination chemotherapy in order to achieve better treatment outcomes (Hu et al., 2010; Tilekar et al., 2020). Trastuzumab in combination with paclitaxel chemotherapy for the treatment of BC was first reported by Slamon et al. (2001). In recent years, Co-NDDSs have been combined with chemotherapy to counter the drawbacks of low bioavailability and poor targeting of chemotherapeutic drugs, thereby enhancing the anti-cancer effects (Xiao et al., 2012). However, they require further research to address the problem of enhanced autophagy levels in tumour cells by chemotherapeutic drugs and thus reduced drug utilisation.

Based on the above background, we successfully prepared a Co-NDDS loaded with the chemotherapeutic drug DOX and the autophagy inhibitor HCQ. The smaller NPs FeAC-DOX NPs was encapsulated with the chemotherapeutic drug DOX, and then FeAC-DOX NPs and the autophagy inhibitor HCQ were co-encapsulated in the larger NPs FeAC-DOX@PC-HCQ NPs. The Co-NDDS is highly biocompatible and effectively improves the bioavailability of the drug, and the prepared NDDS was verified by various assays to have good physicochemical properties and morphological characteristics to compensate for these drawbacks. Among them, FeAC-DOX@PC-HCQ NPs were observed to be uniformly dispersed with a diameter of approximately 200 nm under transmission electron microscopy (TEM); the particle size of the NPs was measured by DLS to be 255.7 ± 18.45 nm, which is similar to the results obtained under TEM, and NPs with this size distribution have enhanced permeability and retention effect (EPR), resulting in accumulation at cancer sites more (Ngoune et al., 2016). Smaller NPs loaded with the chemotherapeutic drug DOX alone measured a particle size of 59.3 ± 7.1 nm, a size suitable for entry into the nucleus through the nuclear pores of BC cells (Fan et al., 2015). The results of the infrared spectroscopy and fluorescence tracing experiments also provide ample evidence that both drugs were successfully encapsulated in the final NPs.

In addition, the release pattern of FeAC-DOX@PC-HCQ NPs was examined by simulating the neutral blood environment and acidic tumour cell environment pH *in vivo*, which showed that the outermost CS and PLGA nanoshells could protect HCQ from releasing less in the blood environment and more in the acidic tumour environment, which might be related to the fact that CS undergoes high dissolution under acidic conditions (Milosavljevic et al., 2020). We also verified that FeAC-DOX NPs is less likely to release drug in the simulated acidic cytoplasmic environment as well as in the lysosomal environment and more in the neutral environment of the nucleus, which may be due to the enhanced binding between CS and ALG in the acidic environment, thus protecting the chemotherapeutic drug DOX inside (Du et al., 2013). In summary, our NDDS effectively avoids the release of drugs at normal physiological sites, instead concentrating on tumour sites, and further enables the release of chemotherapeutic drugs acting on the nucleus of tumour cells in the nucleus rather than in the cytoplasmic environment.

To investigate the *in vitro* cytotoxicity of the drug delivery system, two different human BC cell lines were selected as model cells, including MCF-7 cells representing an *in-situ* ER-positive BC cell line with low malignancy and MDA-MB-231 cells representing a triple negative BC cell with high malignancy (Nordin et al., 2018). By testing the *in vitro* cytotoxicity of the blank nanomaterials at different concentrations, it was well demonstrated that our selected materials have excellent biocompatibility and can be safely used as drug delivery carriers. These results are closely related to the good biocompatibility and low toxicity of materials such as CS and ALG (Li et al., 2022). On the other hand, NDDSs containing the same concentration of the target drug were able to inhibit the survival of BC cells more effectively than free drugs. The autophagy inhibitor HCQ affects the degradation of autophagosomes in the late stages of autophagy by inhibiting the fusion of autophagosomes with lysosomes, thereby enhancing the killing of tumour cells by chemotherapeutic drugs (Dragowska et al., 2013). Equally importantly, the efficient tumour cell killing effect of our designed co-loaded nanosystems is closely related to the reasonable particle size and pH-responsive drug release, thus coping with the complex tumour cell environment. This is further corroborated by the wound healing assay, which also demonstrates that the NDDS can inhibit tumour cell proliferation.

Finally, we examined the expression of autophagy-related proteins by western blotting assay and MDC fluorescence staining of autophagic vesicles to demonstrate that the NDDS can better inhibit autophagy in tumour cells. HCQ affects the degradation of autophagosomes at the late stage of autophagy by inhibiting the fusion of autophagosomes with lysosomes, thus improving the chemotherapeutic effect of tumours (Zhang et al., 2014).

Due to the time and conditions of the study, we have not yet conducted *in vivo* experiments in a tumour-bearing animal model. We will continue to improve and innovate by using *in vivo* tumour-bearing animal models to further validate the efficiency and safety of the *in vivo* antitumour effect and to reduce the side effects of chemotherapy drugs, such as the cardiotoxicity of DOX (Shafei et al., 2017).

## 5 Conclusion

**FIGURE 10:**
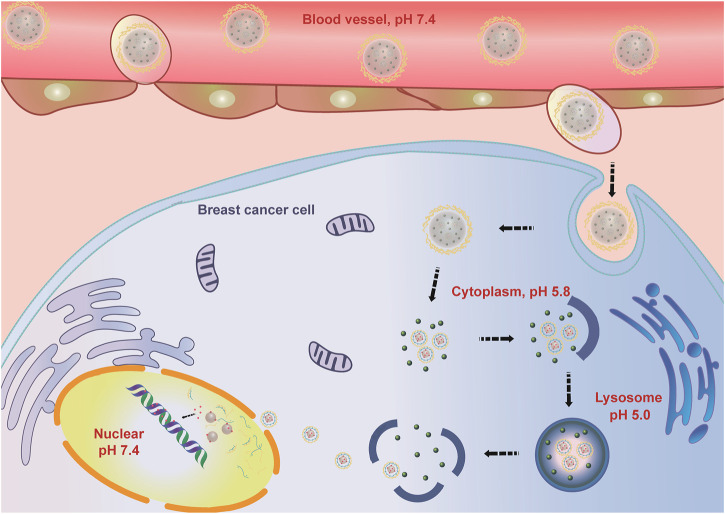
Schematic illustration of action of Co-NDDS.

In this study, a Co-NDDS, FeAC-DOX@PC-HCQ NPs, which can encapsulate the chemotherapeutic drug DOX and the autophagy inhibitor HCQ into their respective domains, was successfully prepared and characterized based on the idea of anti-cancer sensitization. The NDDS consists of smaller internal NPs carrying the chemotherapeutic drug DOX (FeAC-DOX NPs) and the autophagy inhibitor HCQ, with the smaller NPs being loaded into FeAC-DOX@PC-HCQ NPs are released less in the neutral blood environment and more in the acidic tumour environment. release. The drug HCQ prevented late nuclear endosomes from binding to lysosomes and blocked the cellular autophagy process, thus protecting the chemotherapeutic drug DOX from being degraded and destroyed by autophagy. FeAC-DOX NPs entered the nucleus of BC cells through the nuclear pore, and the smaller NPs could release DOX and target DNA in the nucleus under the alkaline environment (pH 7.4), thus exerting pharmacological effects to kill cancer cells (Figure 10). The results show that the Co-NDDS has excellent physicochemical characteristics, and its pH-sensitive property enables precise drug release in BC cells, thus increasing drug delivery to the cytoplasm and nucleus, effectively inhibiting autophagic degradation of tumour cells, and enhancing the cytotoxic effect of anti-cancer drugs on tumour cells. In conclusion, this Co-NDDS could be a promising platform for the treatment of BC.

## Data Availability

The original contributions presented in the study are included in the article/supplementary material further inquiries can be directed to the corresponding authors.
